# Bone Health After Metabolic and Bariatric Surgery: Osteometabolism Biomarkers, Bone Mineral Density, and Microarchitecture

**DOI:** 10.1002/oby.70132

**Published:** 2026-02-17

**Authors:** Camila Medeiros de Almeida, Karynne Grutter Lopes, Michelle da Costa Tavares Bezerra, Paulo Roberto Falcão Leal, Eliete Bouskela, Eduardo Medeiros Ferreira da Gama, Maria Lucia Fleiuss de Farias, Miguel Madeira, Luiz Guilherme Kraemer‐Aguiar

**Affiliations:** ^1^ Postgraduate Program in Clinical and Experimental Physiopathology, Faculty of Medical Sciences State University of Rio de Janeiro Rio de Janeiro Brazil; ^2^ Obesity Unit, Multiuser Clinical Research Center (CePeM), Hospital Universitário Pedro Ernesto State University of Rio de Janeiro Rio de Janeiro Brazil; ^3^ Department of Physiological Sciences, Roberto Alcantara Gomes Institute of Biology State University of Rio de Janeiro Rio de Janeiro Brazil; ^4^ General Surgery, Department of Surgery, Faculty of Medical Sciences State University of Rio de Janeiro Rio de Janeiro Brazil; ^5^ Endocrinology Division Federal University of Rio de Janeiro Rio de Janeiro Brazil; ^6^ Endocrinology, Department of Internal Medicine, Faculty of Medical Sciences State University of Rio de Janeiro Rio de Janeiro Brazil

**Keywords:** bone microarchitecture, bone mineral density, metabolic and bariatric surgery, obesity

## Abstract

**Objective:**

The effects of metabolic and bariatric surgery on bone health still require further investigation. Osteometabolism biomarkers, bone microarchitecture (BM), and mineral density (BMD) of patients who have undergone Roux‐en‐Y gastric bypass (RYGB) within 2–5 years from surgery were compared to non‐surgical age‐, sex‐, and BMI‐matched controls.

**Methods:**

This cross‐sectional study included 39 patients following RYGB (BG: aged = 39 ± 5 years, BMI = 42.2 ± 3.8 kg/m^2^) and 21 controls (CG). Circulating levels of albumin, calcium, phosphorus, magnesium, 25(OH) vitamin D, parathyroid hormone (PTH), carboxy‐terminal telopeptide of type 1 collagen (CTX‐1), and amino‐terminal propeptide of type 1 procollagen (P1NP) were assayed. BMD and BM were assessed by dual‐energy X‐ray absorptiometry (DXA) and three‐dimensional high‐resolution peripheral quantitative computed tomography system (HR‐pQCT).

**Results:**

BG presented with higher circulating phosphorus, PTH, CTX‐1, and P1NP and lower 25(OH) vitamin D compared to CG. DXA parameters did not differ between groups. However, HR‐pQCT revealed significant derangements in BM (trabecular thickness, cortical bone density, and outer trabecular volumetric BMD) in BG. BG showed tibia‐specific trabecular microarchitecture impairment, while sex and BMI showed the expected associations with BM measures.

**Conclusions:**

RYGB was associated with detrimental effects on osteometabolism biomarkers, as well as on density and structural parameters of BM. Early preventive strategies aimed at mitigating these deleterious effects should be systematically evaluated to minimize their long‐term impact.

**Trial Registration:** ClinicalTrials.gov (NCT04193397)

## 1 Introduction

Obesity is a chronic and multifactorial disease that impairs several health outcomes, being a risk factor for musculoskeletal diseases [1]. Metabolic and bariatric surgery (MBS) is an effective treatment for obesity, especially considering the magnitude of weight loss and the improvement of associated comorbidities. Among surgical techniques employed, Roux‐en‐Y gastric bypass (RYGB) is regarded as the gold standard procedure [2, 3, 4, 5].

Typically, obesity is a disease known to act against bone loss, although it can indeed induce vitamin D deficiency, due to its sequestration into the adipose tissue and the resultant reduction of circulating bioavailability [6]. This phenomenon may cause secondary hyperparathyroidism and, consequently, bone damage.

Although MBS provides substantial benefits in terms of weight loss and cardiometabolic outcomes, it also constitutes an additional risk factor for secondary hyperparathyroidism, primarily due to impaired absorption of vitamin D and calcium. Additionally, MBS reduces mechanical load on bones, impacting osteoblast differentiation and bone formation, while changes in hormonal levels following MBS may also interfere with bone health [6, 7, 8]. After MBS, some patients experience expressive reductions in muscle mass, which may contribute to bone mass loss [9]. Importantly, it is already known that muscle mass correlates positively with bone microarchitecture (BM), meaning that as muscle mass decreases [10, 11], bone strength and structure can also be adversely affected. Body composition encompasses separate areas that include adipose tissue, bones, and muscles. Consequently, significant changes in the amount of deposits in one of these compartments will influence the others. All these factors underscore the importance of monitoring and managing bone health in patients undergoing MBS.

Bone mass progressively declines with advancing age. In women, this process accelerates markedly after menopause, primarily due to the abrupt reduction in circulating estrogen—a hormone that plays a critical role in bone homeostasis by simultaneously suppressing bone resorption and stimulating bone formation. Consequently, the menopausal transition is characterized by increased bone loss and a higher risk of osteoporosis, skeletal fragility, and fractures [12, 13]. In line with these findings, some studies showed a precocious reduction in bone mineral density (BMD) just 3 months after MBS, while a progressive increase in bone turnover occurred at 12 months [6, 14]. BM was also negatively influenced in patients with obesity 2 years following RYGB, even after reaching the weight loss plateau [15, 16].

The bone health of patients undergoing MBS deserves to be better studied since the benefits of this surgery for several systems are undeniable. Bone health within 2–5 years after MBS was investigated by comparing osteometabolism biomarkers, BM, and BMD with those of non‐surgical controls. We hypothesize that MBS may exert negative effects on BMD and BM, while alterations in bone turnover biomarkers would indicate ongoing skeletal deterioration even within 2–5 years after this procedure.

## 2 Methods

### 2.1 Patients

We enrolled 330 individuals who had undergone RYGB. Of them, 291 did not meet the eligibility criteria: postmenopausal women (*n* = 2), use of hormone replacement therapy (*n* = 2), cancer or chronic kidney disease (*n* = 2), smoking or alcoholism (*n* = 5), physically active (*n* = 2), alternative bariatric procedure or revisional surgery (*n* = 28), less than 2 or more than 5 years since surgery (*n* = 31), and age 50 years or older (*n* = 78). Additionally, 141 participants were included but did not complete follow‐up. As a result, 39 post‐RYGB patients (92.3% females, aged 39 ± 5 years) composed the Bariatric Group (BG). The Control Group (CG) was composed of 21 BMI‐, age‐, and sex‐matched non‐surgical individuals (Table 1), recruited using snowball sampling via social media, an effective and efficient method for enrolling study participants [17]. BG had a preoperative weight and BMI of 111.4 ± 12.9 kg and 42.2 ± 3.8 kg/m^2^, respectively, as well as an excess weight loss (EWL) after surgery of 104.8% ± 20.5%, a ratio of weight regain (RWR) of 14.1% ± 9.5%, and a mean time since surgery of 3.5 ± 1.0 years.

**TABLE 1:** Demographic characteristics, clinical history, and biochemical parameters of the participants.

Variable	Bariatric group (*n =* 39)	Control group (*n =* 21)	*p* value
Demographic characteristics			
Age (years)	39 ± 5	40 ± 5	0.24
Female, *n* (%)	36 (92.3)	18 (85.7)	0.42
BMI (kg/m^2^)	27.1 ± 3.5	27.6 ± 2.5	0.23
Neck circumference (cm)	32.5 [31.7 ***–*** 34.8]	33 [32.8 ***–*** 35]	0.97
Waist circumference (cm)	82.2 ± 7.7*	87.3 ± 9.6	**0.02**
Hip circumference (cm)	104.2 ± 9.5*	110.2 ± 7.8	**< 0.001**
Systolic blood pressure (mmHg)	113 ± 14*	121 ± 19	**0.04**
Diastolic blood pressure (mmHg)	74 [70–80]	76 [71–90]	0.07
Heart rate (bpm)	71 ± 8	74 ± 11	0.15
Clinical history, *n* (%)			
Type 2 diabetes mellitus	1 (2.6)	2 (9.5)	0.24
Hypertension	1 (2.6)	3 (14.3)	0.09
Hypothyroidism	2 (5.1)	1 (4.8)	0.95
Calcium supplement use	3 (7.7)	0 (0)	0.19
Cholecalciferol supplement use	13 (33)*	0 (0)	**< 0.001**
Osteometabolism biomarkers			
Albumin (g/dL)	4.2 [4.1–4.3]	4.2 [3.9–4.4]	0.57
Calcium (mg/dL)	9.53 ± 0.56	9.60 ± 0.40	0.21
Phosphorus (mg/dL)	3.92 ± 0.44*	3.61 ± 0.35	**0.03**
Magnesium (mg/dL)	2.08 ± 0.17	1.99 ± 0.19	0.46
25(OH) vitamin D (ng/mL)	24.46 ± 8.30*	27.91 ± 6.33	**0.05**
PTH (pg/mL)	80.7 [62.4–118]*	43.2 [28.6–66.9]	**< 0.001**
CTX‐1 (ng/mL)	0.573 [0.391–0.886]*	0.316 [0.197–0.552]	**< 0.001**
P1NP (ng/mL)	94.89 [78.62–114]*	50.35 [40.04–64.22]	**< 0.001**
Vitamin D deficiency (*n*, %)	13 (33.3)	3 (14.2)	0.13
Secondary hyperparathyroidism (*n*, %)	26 (66.6%)*	5 (23.8)	**< 0.001**

*Note*: Results expressed as mean ±SD or median [percentiles 25–75] or n (%). Bold p values indicate statistical significance.

Abbreviations: CTX‐1, carboxy‐terminal telopeptide of type 1 collagen; P1NP, amino‐terminal propeptide of type 1 procollagen; PTH, parathyroid hormone.

* *p* value, unpaired Student *t‐*test or Mann–Whitney test or chi‐square test.

**FIGURE 1:**
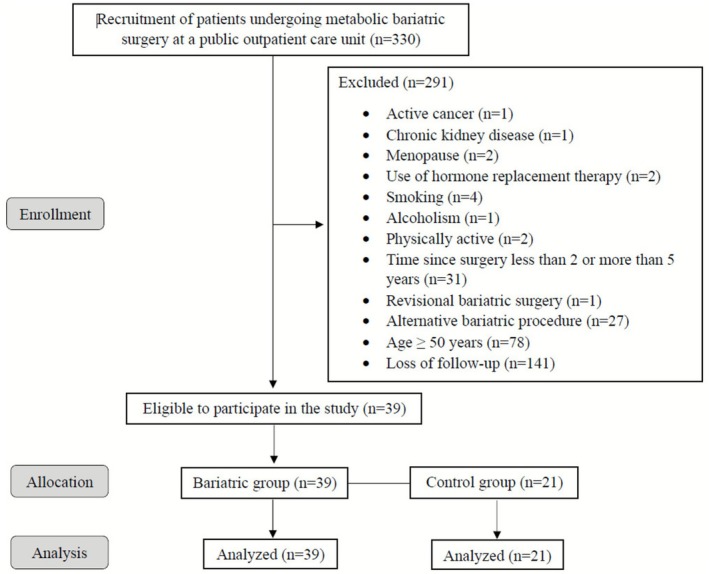
Flowchart illustrating patient screening, enrollment, group allocation, and inclusion in the final analyses.

Inclusion criteria included patients who had undergone RYGB exclusively, were between 18 and 50 years old, and were 2–5 years from surgery. Exclusion criteria included age ≥ 50 years, physical activity level ≥ 150 min/week, cancer, neurological or orthopedic disabilities, chronic kidney, liver, and hematological diseases, musculoskeletal disorders, HIV infection, pregnancy, smoking, alcoholism, menopause/andropause, use of any hormonal replacement therapy or medications affecting bone metabolism, and history of revisional surgery or alternative bariatric procedures. Figure 1 presents the flowchart of the enrollment, allocation, dropouts, follow‐up, and analysis of participants.

### 2.2 Experimental Design and Ethical Approval

The recruitment, pre‐participation screening, allocation, and data collection occurred between September 2023 and September 2024 at a public outpatient care unit. All assessments occurred over 3 days, interspersed with 1‐week intervals, in the following order: (a) visit 1: inclusion and exclusion criteria application, written informed consent, clinical history, physical examination and demographic data, and anthropometric, blood pressure, and heart rate measurements; (b) visit 2: blood samples collection to assess osteometabolism biomarkers; and (c) visit 3: bone microarchitecture and BMD measurements.

This cross‐sectional study was approved by the Ethics Committee of the Pedro Ernesto University Hospital (CAAE 16425419.8.0000.5259). All procedures were performed according to the principles of the Declaration of Helsinki.

### 2.3 Anthropometry

An electronic scale and stadiometer (Welmy W300A, São Paulo, SP, Brazil) measured body mass and height. Body mass index (BMI) was calculated as the weight divided by the height squared (in kg/m^2^). Body circumferences were assessed using a flexible steel measuring tape according to standard procedures. Preoperative and nadir weights were self‐reported during a medical appointment [18]. EWL and RWR were obtained as follows: (a) EWL = (preoperative weight—nadir weight)/(preoperative weight—ideal weight for BMI of 25 kg/m^2^)*100% and (b) RWR = (current weight—nadir weight)/(preoperative weight—nadir weight)*100% [19].

### 2.4 Hemodynamic Measurements

Systolic/diastolic blood pressure (SBP/DBP) and heart rate were assessed by a semiautomated oscillometric device (G‐Tech BSP11, Hangzhou, Zhejiang, China) in a controlled and quiet environment after 10 min of resting in a sitting position, according to standard recommendations [20].

### 2.5 Biochemical Parameters

Blood samples were collected in the morning (07:00–09:00 h) after an overnight fast (≥ 10 h). Analysis of serum levels of calcium, phosphorus, and albumin was performed by colorimetric methods purple phthalein, phosphorus molybdate, and Modified Roy, respectively [21]. The standard adult reference ranges used for these parameters were calcium, 8.6–10.2 mg/dL; phosphorus, 2.7–4.5 mg/dL; and albumin, 3.4–4.8 g/dL. These ranges are typically used when interpreting basic metabolic panels or comprehensive metabolic panels. All analyses were performed using commercially available kits from Labtest (Lagoa Santa, Minas Gerais, Brazil) in an automatic analyzer.

According to a previously published study protocol [22], bone metabolism biomarkers were initially planned to be analyzed using enzyme‐linked immunosorbent assay (ELISA) kits; however, the final analyses were performed using automated immunoassays to improve analytical precision and standardization. Serum concentrations of amino‐terminal propeptide of type 1 procollagen (P1NP), carboxy‐terminal telopeptide of type 1 collagen (CTX‐1), parathyroid hormone (PTH), and 25‐hydroxyvitamin D (25[OH]D) were measured using the MAGLUMI Total chemiluminescence immunoassay (CLIA; Shenzhen New Industries Biomedical Engineering Co. Ltd., Shenzhen, P.R. China), as follows: P1NP: catalog #130211005M; lower limit of detection (LoD): 3.0 ng/mL; intra‐assay coefficient of variation (CV): < 3.3%; inter‐assay CV: < 4.9%; CTX‐1: catalog #130211006M; LoD: 0.010 ng/mL; intra‐assay CV: ≤ 4.3%; inter‐assay CV: ≤ 5.7%; PTH: catalog #130261001M; LoD: 3.0 pg/mL; intra‐assay CV: ≤ 3.1%; inter‐assay CV: ≤ 5.1%; 25(OH)D: catalog #130261004M; LoD: 0.50 ng/mL; intra‐assay CV: ≤ 4.2%; inter‐assay CV: ≤ 6.8%. All measurements were performed on a Cobas e601 analyzer (Roche Diagnostics GmbH, Mannheim, Germany). All the assays were performed in duplicate in a single analytical run using reagents from the same lot. Quality control sera provided by the manufacturer were used in each run, and results met the manufacturer's quality specifications. The reference ranges for bone turnover and related endocrine markers were: P1NP, 15–59 ng/mL; CTX‐1, 0.137–0.573 and 0.142–0.584 ng/mL, for women and men, respectively; PTH, 15–65 pg/mL; and 25(OH)D, 30–100 ng/mL. The lower limits of detection were: 3.00 pg/mL for PTH; 0.50 ng/mL for 25(OH) D; 3.00 ng/mL for P1NP; and 0.010 ng/mL for CTX‐1.

### 2.6 High‐Resolution Peripheral Quantitative Computed Tomography (HR‐pQCT)

Volumetric bone mineral density (vBMD) and BM were assessed using a three‐dimensional first generation HR‐pQCT (XtremeCT, Scanco Medical AG, Brüttisellen, Switzerland), analyzing nondominant distal radius and tibia, appropriately immobilized on an anatomically formed carbon fiber shell [23]. A two‐dimensional detector coupled with a 0.08‐mm point‐focus X‐ray source enables this system to acquire multiple CT slices with a nominal resolution of 82 μm. The reference line is manually positioned at the end plate of the radius and tibia using anteroposterior scout views, with the first CT slice at the distal radius and distal tibia positioned 9.5 and 22.5 mm proximal to the reference line, respectively. At each site, 110 consecutive slices were obtained, providing a three‐dimensional image of approximately 9 mm in the axial plane. The radiation exposure was comparable to that of standard dual‐energy X‐ray absorptiometry (DXA), remaining below 3 μSv per measurement. Attenuation data were converted to equivalent hydroxyapatite (HA) densities. All images were visually inspected for artifacts, and scans affected by motion or other distortions were excluded. Image analyses were performed by the same certified technician throughout the study. The reproducibility of density‐based measures is generally less than 1% and between 3% and 5% for bone structure parameters [24].

We analyzed the percentage of trabecular bone volume (BV/TV), trabecular number (Tb.N), trabecular thickness (Tb.Th), trabecular separation (Tb.Sp), standard deviation of trabecular separation (Tb.Sp 1/*N* SD), cortical thickness (Ct.Th), cortical perimeter (Ct.Pm), total bone area (Tt.Ar), cortical area (Ct. Ar), trabecular area (Tb.Ar), volumetric bone density at entire bone (Tt.BMD), cortical bone density (Ct.BMD), trabecular bone density (Tb.BMD), meta trabecular bone density (Dmeta), inner trabecular bone density (Dinn), and meta to inner bone density ratio (Meta/Inn).

### 2.7 Dual‐Energy X‐Ray Absorptiometry (DXA)

Body composition (body fat, fat‐free mass, bone mineral content, and lean mass) and areal BMD were assessed by DXA (Prodigy‐GE Lunar Prodigy Advance, GE Healthcare Madison, WI, USA). BMD at the lumbar spine (L1–L4), femoral neck, total femur, and one‐third radius was expressed in absolute values (g/cm^2^) and as standard deviations (SD) from the expected BMD for the age‐matched population. According to the 2019 Official Positions of the International Society for Clinical Densitometry, *z*‐score ≤ −2.0 SD is a diagnosis of low bone mass for age, while above −2.0 is within the expected range for the age period. All DXA measurements were performed by the same experienced technologist [10].

### 2.8 Statistical Analysis

Normal distribution was investigated by the Shapiro–Wilk test. Between‐group differences were tested through unpaired Student *t‐*test or Mann–Whitney, and the results were expressed as mean ± SD or median (percentiles 25–75). Based on the biological relevance of metabolic bone changes, Tb.Th at the distal radius and Ct.BMD at the distal tibia were designated as the primary endpoints. All other HR‐pQCT parameters, including structural and density measures at both sites, were considered secondary. Statistical power calculations were performed using the expected differences in the primary endpoints, with *α* = 0.05. To account for multiple comparisons across HR‐pQCT parameters and skeletal sites, the Benjamini–Hochberg false discovery rate (FDR) correction was applied. Both unadjusted *p* values and adjusted *q* values are reported. Statistical significance was set at *q* < 0.05. Chi‐square tests were used for categorical data and presented as frequency (count and proportion). Pearson or Spearman correlation coefficients were computed to examine the associations between MBS data (time since surgery, EWL, and RWR), bone metabolism biomarkers (P1NP, CTX‐1, PTH, and 25[OH]D), and HR‐pQCT parameters. To examine the independent associations between MBS and bone parameters, multivariable linear regression models were constructed for bone outcomes at the radius and tibia (Tt.BMD, Ct.BMD, Tb.BMD, and Tb.Th). The main exposure variable was group (BG = 1, CG = 0). All models were adjusted for age, sex, BMI, time since surgery, serum 25(OH)D, and PTH levels. Results are presented as standardized β coefficients (β_std) with corresponding 95% confidence intervals (CI) and *p* values. All calculations were analyzed using NCSS statistical software version 10 (Kaysville, UT, USA), and the statistical significance was set at *p* ≤ 0.05.

## 3 Results

Demographic characteristics, clinical history, and metabolic profile of patients are exhibited in Table 1. The groups were similar for many variables, except for waist/hip circumference and SBP, which were lower in the BG group compared to CG (*p ≤* 0.04). The use of vitamin D supplements was more frequent in BG than in CG (*p <* 0.001). In addition, BG presented with higher phosphorus, PTH, CTX ‐1, and P1NP and lower 25(OH)D levels than CG (*p ≤* 0.05). Among these parameters, only PTH and P1NP exhibited levels above the reference. The 25(OH)D deficiency (< 20 ng/mL) was observed in 13 (33.3%) participants of BG and in 3 (14.2%) of CG (*p =* 0.13). Elevated PTH levels (> 65 pg/mL) were found in 26 (66.6%) patients of BG and 5 individuals (23.8%) of CG (*p <* 0.001).

Table 2 presents bone microarchitecture assessed at two peripheral sites, the tibia and the radius. At the radius, the groups were similar for nearly all structural and density parameters, except for Tb.Th and Dmeta, which were significantly lower in BG than in CG (*q* = 0.01). Likewise, Ct.BMD at the distal tibia was also significantly reduced in BG compared with CG.

**TABLE 2:** Bone microarchitecture expressed as structural and density parameters at the distal radius and distal tibia of the participants.

Variable	Bariatric group (*n =* 39)	Control group (*n =* 21)	*p* value	*q* value (FDR‐adjusted)
Distal radius—structural parameters				
BV/TV (%)	0.116 ± 0.032*	0.140 ± 0.037	**0.02**	0.09
Tb.N (mm^−1^)	1.880 ± 0.261	1.927 ± 0.290	0.54	0.66
Tb.Th (mm)	0.060 [0.051–0.068]* ^,^ ^a^	0.072 [0.060–0.084]	**< 0.001**	**0.01**
Tb.Sp (mm)	0.464 [0.422–0.533]	0.444 [0.393–0.513]	0.21	0.39
Tb.Sp 1/*N* SD (mm)	0.202 [0.169–0.239]	0.179 [0.162–0.210]	0.28	0.47
Ct.Th (mm)	0.825 ± 0.196	0.915 ± 0.183	0.10	0.22
Ct.Pm (mm)	68.1 ± 7.9	65.3 ± 9.9	0.26	0.46
Tt.Ar (mm^2^)	258.3 ± 51.3	243.1 ± 72.1	0.38	0.54
Ct.Ar (mm^2^)	55.5 ± 11.6	58.9 ± 10.4	0.30	0.48
Tb.Ar (mm^2^)	195.7 ± 53.3	181.2 ± 68.9	0.39	0.54
Distal radius—density parameters				
Tt.BMD (mg HA/cm^3^)	336.9 ± 81.6*	391.8 ± 74.1	**0.02**	0.09
Ct.BMD (mg HA/cm^3^)	940.5 ± 61.2*	979.7 ± 62.3	**0.03**	0.09
Tb.BMD (mg HA/cm^3^)	139.9 ± 39.1*	168.5 ± 45.5	**0.02**	0.09
Dmeta (mg HA/cm^3^)	200.7 ± 36.1* ^,^ ^a^	231.7 ± 40.5	**< 0.001**	**0.01**
Dinn (mg HA/cm^3^)	97.9 ± 41.9*	124.7 ± 50.4	**0.04**	0.11
Meta/Inn (−)	2.13 [1.82–2.55]	1.85 [1.59–2.45]	0.14	0.29
Distal tibia—structural parameters				
BV/TV (%)	0.117 ± 0.030	0.132 ± 0.037	0.09	0.22
Tb.Th (mm)	0.064 ± 0.014*	0.073 ± 0.014	**0.02**	0.09
Tb.N (mm^−1^)	1.83 ± 0.31	1.82 ± 0.36	0.84	0.92
Tb.Sp (mm)	0.497 ± 0.096	0.500 ± 0.119	0.92	0.95
Tb.Sp 1/*N* SD (mm)	0.211 [0.176–0.262]	0.220 [0.179–0.276]	0.89	0.94
Ct.Th (mm)	1.207 ± 0.249	1.264 ± 0.191	0.38	0.54
Ct.Pm (mm)	102.9 ± 7.5	100.8 ± 12.6	0.43	0.57
Tt.Ar (mm^2^)	692.9 ± 92.8	668.4 ± 156.6	0.48	0.61
Ct.Ar (mm^2^)	120.7 [111.3–141.1]	119.5 [115.5–137.2]	0.82	0.92
Tb.Ar (mm^2^)	559.4 ± 100.5	539.9 ± 151.1	0.58	0.68
Distal tibia—density parameters				
Tt.BMD (mg HA/cm^3^)	279.1 [248.5–329.5]*	311.9 [284.7–369.2]	**0.03**	0.09
Ct.BMD (mg HA/cm^3^)	934.5 ± 54.1* ^,^ ^a^	974.9 ± 45.1	**< 0.001**	**0.01**
Tb.BMD (mg HA/cm^3^)	140.2 ± 36.9	159.5 ± 44.9	0.09	0.22
Dmeta (mg HA/cm^3^)	201.8 ± 40.7*	227.7 ± 43.9	**0.03**	0.09
Dinn (mg HA/cm^3^)	98.3 ± 36.9	113.2 ± 46.8	0.20	0.39
Meta/Inn (−)	2.15 [1.74–2.55]	2.05 [1.75–2.60]	0.99	0.99

*Note*: Results are expressed as mean ± SD or median [percentiles 25–75]. Bold *p* or *q* values indicate statistical significance.

Abbreviations: BV/TV, percentage of trabecular bone volume; Ct.Ar, cortical area; Ct.BMD, cortical bone density; Ct.Pm, cortical perimeter; Ct.Th, cortical thickness; Dinn, inner trabecular bone density; Dmeta, meta trabecular bone density; HA, hydroxyapatite; Meta/Inn, meta to inner bone density ratio; Tb.Ar, trabecular area; Tb.BMD, trabecular bone mineral density; Tb.N, trabecular number; Tb.Sp 1/*N* SD, SD of TbSp; Tb.Sp, trabecular separation; Tb.Th, trabecular thickness; Tt.Ar, total area; Tt.BMD, volumetric bone density at entire bone.

* *p* value, unpaired Student *t‐*test or Mann–Whitney test.

^a^ *q* value, after FDR correction (Benjamini–Hochberg).

Body composition parameters, including fat mass, fat‐free mass, bone mineral content, and lean mass, did not differ between groups. Even showing important damage in the microarchitecture in individuals who had undergone RYGB, the investigation of areal BMD by DXA had similar results between groups in all assessed sites (such as lumbar spine, femoral neck, total femur, and radius 33%) (Table 3).

**TABLE 3:** Body composition and densitometric data of the participants.

Variable	Bariatric group (*n =* 39)	Control group (*n =* 21)	*p* value
Body composition			
Fat mass (kg)	24.8 [20.4–28.9]	27.4 [22.7–35.2]	0.06
Fat‐free mass (kg)	46 [43.9–50.2]	43.5 [40.7–49]	0.32
Bone mineral content (kg)	2.4 ± 0.3	2.5 ± 0.4	0.33
Lean mass (kg)	43.8 [41.7–47.7]	40.9 [38.3–46.4]	0.30
Densitometric data			
Lumbar spine			
LS BMD (g/cm^2^)	1.158 ± 0.150	1.199 ± 0.143	0.32
LS *z*‐score	−0.356 ± 1.245	−0.252 ± 1.189	0.65
Proximal femur			
Femoral neck BMD (g/cm^2^)	1.030 ± 0.132	1.026 ± 0.107	0.19
Femoral neck *z*‐score	0.131 ± 0.954	−0.014 ± 0.717	0.70
Total femur BMD (g/cm^2^)	1.040 ± 0.136	1.028 ± 0.109	0.30
Total femur *z*‐score	0.184 ± 1.029	0.162 ± 0.865	0.45
Radius 33%			
Radius 33% BMD (g/cm^2^)	0.882 ± 0.079	0.888 ± 0.050	0.75
Radius 33% *z*‐score	−0.200 ± 0.928	−0.165 ± 0.534	0.71

*Note: p* value, unpaired Student *t‐*test or Mann–Whitney test; results are expressed as mean ± SD or median [percentiles 25–75].

Abbreviation: BMD, bone mineral density.

After correction for multiple testing, EWL remained negatively associated with Tb.N at both the distal tibia and radius (*r* = −0.42 and *r* = −0.46, *p <* 0.001), as well as Dmeta of distal tibia (*r* = −0.46, *p <* 0.001). No association between time since surgery and RWR with HR‐pQCT parameters remained significant after correction. Serum CTX‐1 was inversely associated with Ct.BMD at the distal tibia (rho = −0.46, *q* = 0.03). No significant associations were observed between P1NP, PTH, and 25(OH)D and HR‐pQCT parameters.

After multivariable adjustment, surgery was significantly associated with higher Tb.Th at the tibia (β_std = 1.75; 95% CI 0.18 to 3.31; *p =* 0.03). For the remaining HR‐pQCT parameters, the group effect did not reach statistical significance (*p ≥* 0.14). Among covariates, female sex was associated with lower tibial Tt.BMD (β_std = −1.58; 95% CI −3.14 to −0.02; *p =* 0.04) and lower tibial Tb.Th (β_std = −1.96; 95% CI −3.52 to −0.39; *p =* 0.01). Conversely, higher BMI was positively associated with tibial Ct.BMD (β_std =0.42; 95% CI 0.08 to 0.76; *p =* 0.01). In this analysis, age, time since surgery, 25(OH)D, and PTH showed no consistent statistically significant associations with any of the bone outcomes.

## 4 Discussion

This study evaluated bone health in patients who had undergone RYGB 2–5 years earlier, compared with BMI‐, age‐, and sex‐matched non‐surgical controls. Our findings suggest that RYGB may accelerate bone remodeling and contribute to the deterioration of both volumetric BMD and bone microarchitecture. It is worth noting that bone health was assessed using complementary methods, allowing a comprehensive evaluation of osteometabolism biomarkers, particularly those related to bone remodeling, as well as density and structural bone parameters tested by HR‐pQCT.

In contrast with Yu et al. [25], but consistent with other studies [15, 26, 27, 28], we observed higher PTH levels in the BG group. Additionally, 26 out of 39 participants in BG exhibited secondary hyperparathyroidism (PTH > 65 pg/mL) whereas only 5 of the 21 participants in the CG group presented with this condition, particularly deleterious to bone health. Although the prevalence of vitamin D deficiency did not differ significantly between groups, there was a trend toward lower 25(OH)D levels in the BG group, despite higher cholecalciferol supplementation—some participants reported taking more than 2000 IU per day. In contrast, none of the CG participants reported calcium or vitamin D supplementation. These findings emphasize the challenge of maintaining adequate 25(OH)D levels even with supplementation [29]. Notably, fewer than 10% of BG participants reported calcium supplementation, and approximately one‐third used vitamin D supplements. Thus, our findings likely reflect both impaired calcium and vitamin D absorption and suboptimal supplementation—factors known to contribute to bone loss after MBS [8]. This underscores the importance of long‐term follow‐up extending beyond the period of rapid weight loss and into weight‐stable phases, with close attention to adequate vitamins and micronutrients supplementation and bone health maintenance.

In line with others, we noticed higher circulating levels of the biomarkers of bone formation (P1NP) and resorption (CTX) in the BG group compared to CG. We can speculate that the elevated PTH levels observed in the BG group may partially explain these findings, which are consistent with previous prospective studies [15, 26, 30]. Some of these studies also noted a more prominent increase in CTX levels compared to P1NP. Lindeman et al. conducted a prospective study following 21 individuals during 5 years after RYGB and reported results comparable to ours, despite the cross‐sectional design of our study, with persistently elevated CTX and P1NP levels throughout the follow‐up period [31]. These results align with those by Valderas et al., who found elevated CTX and PTH levels in postmenopausal women [27]. Notably, women in our cohort, who were of reproductive age, with preserved gonadotropic function and without other fracture risk factors, showed findings like those observed in postmenopausal women.

As hypothesized, we observed significant deterioration of volumetric BMD and bone microarchitecture in those who underwent RYGB, regardless of the skeletal site assessed. At the distal radius, the BG group exhibited poorer trabecular microarchitecture than the CG group, as indicated by lower Tb.Th and Dmeta. Others have already observed that elevations of PTH levels are consistent with the worsening in radius microstructure, particularly with the alteration concerning cortical bone [32]. Additionally, Ct.BMD at the distal tibia was significantly lower in the BG group than in the CG group. These findings corroborate previous studies that demonstrated microstructural deterioration after MBS, with reduced vBMD at total, cortical/trabecular bone, and Tb.Th, as well as increased Tb.Sp and Tb.Sp 1/*N* SD [15, 26, 28]. Such microarchitectural changes may contribute to the increased fracture risk reported in this population [31, 33]. However, none of our patients reported a previous history of clinical fractures.

In contrast to the findings reported by Frederiksen et al. [30], we observed greater microarchitectural impairment at the distal radius than at the distal tibia, despite the former being a non‐weight‐bearing site. Although previous studies have suggested that reductions in mechanical loading following weight loss contribute to bone loss [8, 26, 34], our results—even within the limits of a cross‐sectional design—do not support this mechanism as the primary driver of the alterations observed in the present analysis. This is consistent with the prospective findings of Diniz‐Souza et al. [35]. While persistently elevated PTH levels could theoretically contribute to preferential deterioration at this predominantly cortical site, no significant correlation was observed between PTH concentrations and the HR‐pQCT parameters in our study population.

Bone loss after MBS therefore appears to result from multiple physiological mechanisms, including malabsorption and inadequate nutritional supplementation (particularly calcium and vitamin D), reductions of lean mass, and hormonal alterations, such as changes in gut peptides secreted by enteroendocrine cells of the gastrointestinal system [36, 37, 38]. The reduction of leptin levels following weight loss may downregulate osteoblast activity while enhancing osteoclastic activity [39]. Gut‐derived peptides, including peptide YY, glucose‐dependent insulinotropic polypeptide, insulin, and ghrelin, also play a role in the regulation of bone remodeling. In summary, anatomical and functional changes of the gastrointestinal tract induced by RYGB alter the secretion of gut peptides, which contributes to increased bone turnover and progressive bone loss [7, 40].

Despite consistent evidence of microarchitectural deterioration, we did not detect differences in areal BMD between groups when assessed by DXA, a finding consistent with a previous retrospective cohort that evaluated lumbar spine and femoral neck BMD [27]. However, our study also found no reduction in the 33% radius aBMD in the BG group, despite a higher prevalence of secondary hyperparathyroidism in this group. The absence of a significant difference at the 33% radius site likely reflects the combined influence of DXA's limited sensitivity to cortical microstructural changes [41]. Nevertheless, the observed microarchitectural deterioration in our study supports the occurrence of true bone impairments at this predominantly cortical site.

In contrast with findings of Yu et al. [25], our DXA results do not appear to represent an artifactual error, a phenomenon that typically arises from changes in body composition, such as significant fat mass reduction or the presence of an adipose panniculus, which can lead to an apparent decrease in BMD. Because of the cross‐sectional design of our study, loss of surrounding soft tissue mass was not evaluated; therefore we cannot confirm whether this mechanism affected DXA beam attenuation and edge detection, potentially introducing an artifactual bias. A longitudinal assessment might have revealed progressive reductions in BMD in the BG group, as reported in previous studies [15, 26, 42]. Finally, potential errors related to patient positioning were minimized, as all DXA scans were performed by a qualified technician following standardized procedures.

Notably, deterioration of bone health in the BG group was evident and paralleled by alterations in bone remodeling markers. If we had opted to investigate bone health solely through DXA, the gold standard technique for assessment of bone mass, these differences would have been overlooked. Our findings highlight the importance of implementing preventive strategies for bone health in the regular follow‐up of a MBS, such as adequate calcium and vitamin D supplementation and exercise counseling focused on preserving lean mass, even in the presence of normal densitometric results. Given that HR‐pQCT is unavailable for most patients, the role of osteometabolism biomarkers is especially important, as they may also provide clinically meaningful signals of bone deterioration when advanced imaging cannot be performed.

Some of our limitations need to be acknowledged. The cross‐sectional design of the study hampers conclusions regarding causal relationships. Our results are limited to the main characteristics of the patients studied, the type of surgery technique employed, and the time elapsed since surgery. Although participants' physical activity level was carefully verified, dietary intake and sun exposure could not be reliably quantified in this cohort, which limits our ability to account for the potential influence of these factors on our findings. Morphometric vertebral analysis using DXA‐based vertebral fracture assessment (VFA), which can detect early and asymptomatic vertebral fractures, was not performed. A considerable proportion of participants were lost to follow‐up due to challenges in maintaining contact—a situation commonly encountered in our setting, as many participants come from socioeconomically disadvantaged communities. Although this reflects a particular reality of our cohort, such attrition is also frequently observed in observational studies of MBS, as patients often transition to external healthcare providers or discontinue follow‐up once weight loss has stabilized. Finally, in those patients who had undergone RYGB, the potential impact of presurgical obesity on bone health should be acknowledged.

## 5 Conclusion

Our study provides evidence that both density and structural parameters of BM are compromised in young patients 2–5 years after RYGB, even when areal BMD assessed by DXA remains within the normal range. In parallel with these subtle structural alterations, osteometabolism biomarkers were also dysregulated. These findings underscore the need for a comprehensive assessment of bone health both before and throughout long‐term follow‐up after MBS, to mitigate bone loss and reduce fracture risk and its associated morbidity. Future studies are required to clarify the physiological mechanisms underlying RYGB‐induced bone deterioration and to further explore the potential of HR‐pQCT as a clinical tool for bone evaluation.

## Author Contributions

Conception and design of the study: Luiz Guilherme Kraemer‐Aguiar. Acquisition, analysis, and interpretation of data: Camila Medeiros de Almeida, Karynne Grutter Lopes, Michelle da Costa Tavares Bezerra, Paulo Roberto Falcão Leal, Eliete Bouskela, and Eduardo Medeiros Ferreira da Gama. Drafting the manuscript: Camila Medeiros de Almeida and Karynne Grutter Lopes. Revising the manuscript: Maria Lucia Fleiuss de Farias, Miguel Madeira, Karynne Grutter Lopes, and Luiz Guilherme Kraemer‐Aguiar. Final revision of the manuscript: Luiz Guilherme Kraemer‐Aguiar. All authors approved the final version of the manuscript.

## Funding

This research was funded by the Carlos Chagas Filho Foundation for Research Support in the State of Rio de Janeiro (FAPERJ), by the National Council for Scientific and Technologic Development (CNPq), and by the Coordination for the Improvement of Higher Education Personnel (CAPES).

## Conflicts of Interest

The authors declare no conflicts of interest.

## Data Availability

The data that support the findings of this study are available from the corresponding author upon reasonable request.
